# Expression of the neuroprotective slow Wallerian degeneration (*Wld*^*S*^) gene in non-neuronal tissues

**DOI:** 10.1186/1471-2202-10-148

**Published:** 2009-12-16

**Authors:** Thomas M Wishart, David G Brownstein, Derek Thomson, Anca M Tabakova, Katherine M Boothe, Jack W Tsao, Thomas H Gillingwater

**Affiliations:** 1 Centre for Integrative Physiology & Euan MacDonald Centre for Motor Neuron Disease Research, University of Edinburgh Medical School, Edinburgh, EH8 9XD, UK; 2 Research Animal Pathology Core Laboratory, Queen's Medical Research Institute, University of Edinburgh, Edinburgh, EH16 4TJ, UK; 3 Department of Neurology, Uniformed Services University of the Health Sciences, Bethesda, MD 20814, USA

## Abstract

**Background:**

The slow Wallerian Degeneration (*Wld*^*S*^) gene specifically protects axonal and synaptic compartments of neurons from a wide variety of degeneration-inducing stimuli, including; traumatic injury, Parkinson's disease, demyelinating neuropathies, some forms of motor neuron disease and global cerebral ischemia. The *Wld*^*S *^gene encodes a novel Ube4b-Nmnat1 chimeric protein (Wld^S ^protein) that is responsible for conferring the neuroprotective phenotype. How the chimeric Wld^S ^protein confers neuroprotection remains controversial, but several studies have shown that expression in neurons *in vivo *and *in vitro *modifies key cellular pathways, including; NAD biosynthesis, ubiquitination, the mitochondrial proteome, cell cycle status and cell stress. Whether similar changes are induced in non-neuronal tissue and organs at a basal level *in vivo *remains to be determined. This may be of particular importance for the development and application of neuroprotective therapeutic strategies based around *Wld*^*S*^-mediated pathways designed for use in human patients.

**Results:**

We have undertaken a detailed analysis of non-neuronal *Wld*^*S *^expression in *Wld*^*S *^mice, alongside gravimetric and histological analyses, to examine the influence of *Wld*^*S *^expression in non-neuronal tissues. We show that expression of *Wld*^*S *^RNA and protein are not restricted to neuronal tissue, but that the relative RNA and protein expression levels rarely correlate in these non-neuronal tissues. We show that *Wld*^*S *^mice have normal body weight and growth characteristics as well as gravimetrically and histologically normal organs, regardless of Wld^S ^protein levels. Finally, we demonstrate that previously reported *Wld*^*S*^-induced changes in cell cycle and cell stress status are neuronal-specific, not recapitulated in non-neuronal tissues at a basal level.

**Conclusions:**

We conclude that expression of Wld^S ^protein has no adverse effects on non-neuronal tissue at a basal level *in vivo*, supporting the possibility of its safe use in future therapeutic strategies targeting axonal and/or synaptic compartments in patients with neurodegenerative disease. Future experiments determining whether Wld^S ^protein can modify responses to injury in non-neuronal tissue are now required.

## Background

Degeneration of axonal and/or synaptic compartments of neurons is an early and pathologically important process in many disorders of the human nervous system, ranging from Alzheimer's disease and Batten disease through to multiple sclerosis and motor neuron disease [1-8]. Therapies designed to specifically delay or halt the progression of axonal and synaptic degeneration are therefore actively being sought for a wide range of neurological disorders.

The most robust delay in axonal and synaptic degeneration reported to date in animal models of neurological disorders has been generated by the introduction of the slow Wallerian degeneration (*Wld*^*S*^) gene. To date, the *Wld*^*S *^gene has been shown to significantly modify disease onset and/or progression in animal models of traumatic axonal injury [9,10], Parkinson's disease [11,12], demyelinating neuropathies [13], some forms of motor neuron disease [14] and cerebral ischemia [15]. These experiments highlight the potential for using the Wld^S ^protein and/or its downstream molecular interactions to generate novel therapeutic approaches for the treatment of neurological disorders. Importantly, the ability to successfully deliver the *Wld*^*S *^gene and confer robust neuroprotection using gene therapy approaches [16,17] has opened up the possibility of directly delivering *Wld*^*S*^-related therapies to human patients.

The chimeric *Wld*^*S *^gene occurred as the result of a spontaneous mutation in the C57BL/6 line of mice (originally termed C57BL/6/Ola [9]), resulting in a tandem triplication of a region already present on the distal region of chromosome 4. Mice carrying the *Wld*^*S *^mutation are otherwise indistinguishable from their C57BL/6J strain mates in genotyping of more than 50 microsatellite markers and restriction fragment length polymorphisms (RFLPs [18-20]). The triplicated region contains sequences coding for Nmnat1, Rbp7 and Ube4b [21]. The boundaries within the triplicated region result in 2 copies of a fusion gene comprising the N70 terminal amino acids of Ube4b and the entire coding region of Nmnat1 (C Terminal 285 amino acids), linked by 18 amino acids from the 5' untranslated region of Nmnat1 which are not normally expressed [18,21,22]. The chimeric portion of the triplication (i.e. the N-70 Ube4b/Nmnat1 C-303 chimera) has been shown to be sufficient to recapitulate the *Wld*^*S *^phenotype through the generation of transgenic lines in mice, rats and drosophila [23-25].

Although the *Wld*^*S *^gene has obvious therapeutic potential, its mechanism of action remains unclear. However, several studies have shown that expression of Wld^S ^protein in neurons *in vivo *and *in vitro *induces changes in core cellular pathways, including; NAD biosynthesis, ubiquitination, the mitochondrial proteome, cell cycle status and cell stress [17,26-28]. Even though the extent to which each of these modifications contribute to the neuroprotective phenotype remains unclear, the fact that *Wld*^*S *^modifies key cellular pathways raises potential problems for its use as a therapeutic agent that have yet to be investigated. For example, it is conceivable that changes in NAD biosynthesis pathways and/or cell cycle pathways in non-neuronal organs and tissues may alter their form and/or function.

Here, we have undertaken the first detailed study of the effects of *Wld*^*S *^expression in non-neuronal tissues *in vivo*. We show that Wld^S ^protein is expressed at differing levels in a range of non-neuronal organs in *Wld*^*S *^mice. Systemic expression of *Wld*^*S *^did not affect overall body weight or growth. Gravimetric and histological analysis of a wide range of organs and tissues confirmed no changes in *Wld*^*S *^mice. We also demonstrate that previously reported alterations in cell cycle and cell stress proteins reported in *Wld*^*S *^brain tissue are a neuronal-specific response not observed in non-neuronal organs *in vivo*.

## Results

### *Wld*^*S *^expression is not limited to neuronal tissue in vivo

**Figure 1:**
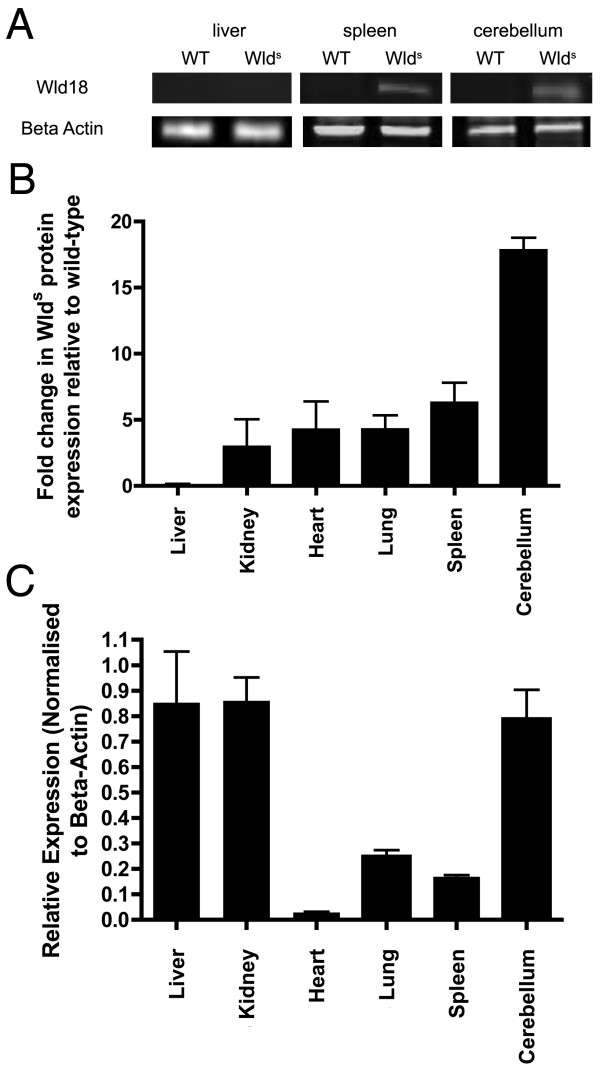
**Expression of Wld^S ^protein and mRNA in non-neuronal tissue**. A - Representative examples of bands obtained with quantitative fluorescent western blots from wild-type and *Wld*^*S *^mouse liver, spleen and cerebellum probed with antibodies against Wld^S ^protein (Wld-18) and beta-actin (loading control). B - Wld^S ^protein levels in organs from *Wld*^*S *^mice expressed as fold change in protein relative to wild-type tissue. Note that all organs except for the liver showed significant levels of Wld^S ^protein, but none to the same magnitude as found in the cerebellum. C - *Wld*^*S *^RNA levels in organs from *Wld*^*S *^mice shown as relative expression normalised to wild-type. Note that RNA levels did not always match protein expression levels (c.f. Panel A). A minimum of 3 mice per genotype were used for all experiments.

Despite numerous reports in the literature concerning the therapeutic potential of the *Wld*^*S *^gene (see background above), no previous studies have undertaken a rigorous assessment of the consequences of *Wld*^*S *^expression in non-neuronal tissue. We first examined the expression of Wld^S ^protein and RNA levels in a variety of organs from *Wld*^*S *^mice and wild-type controls and compared expression levels to those observed in the cerebellum [29,30]. Organs examined included liver, kidney, heart, lung and spleen. We used quantitative fluorescent western blotting techniques to assess and compare protein expression levels using a proven antibody for the Wld^S ^protein (Wld-18 antibody [23,30,31]; Figure 1). Example bands are shown in Figure 1A for liver (which had little to no Wld^S ^protein expression), spleen (intermediate levels of expression) and the cerebellum (strong expression).

These experiments showed that Wld^S ^protein expression was not limited to neuronal tissue, but that it was not strongly expressed in all organs examined (Figure 1B). For example, the liver had the lowest expression at 0.06 ± 0.09 (mean ± SEM) fold increase compared to wild-type background signal, the spleen showed a 6.27 ± 1.05 fold increase, whereas the cerebellum showed a 17.82 ± 0.96 fold increase. We also examined RNA expression in *Wld*^*S *^mice (Figure 1C) and found that RNA expression varied greatly between different organs and did not necessarily correlate with protein expression. For example, the liver (which showed the lowest protein expression of the organs examined) had relatively high RNA expression levels, approaching those observed in the cerebellum. Taken together, these findings demonstrate that Wld^S ^protein and RNA are present in a range of non-neuronal organs at differing levels, but also demonstrate that the presence of RNA does not always indicate that Wld^S ^protein will also be present at comparable levels.

**Figure 2:**
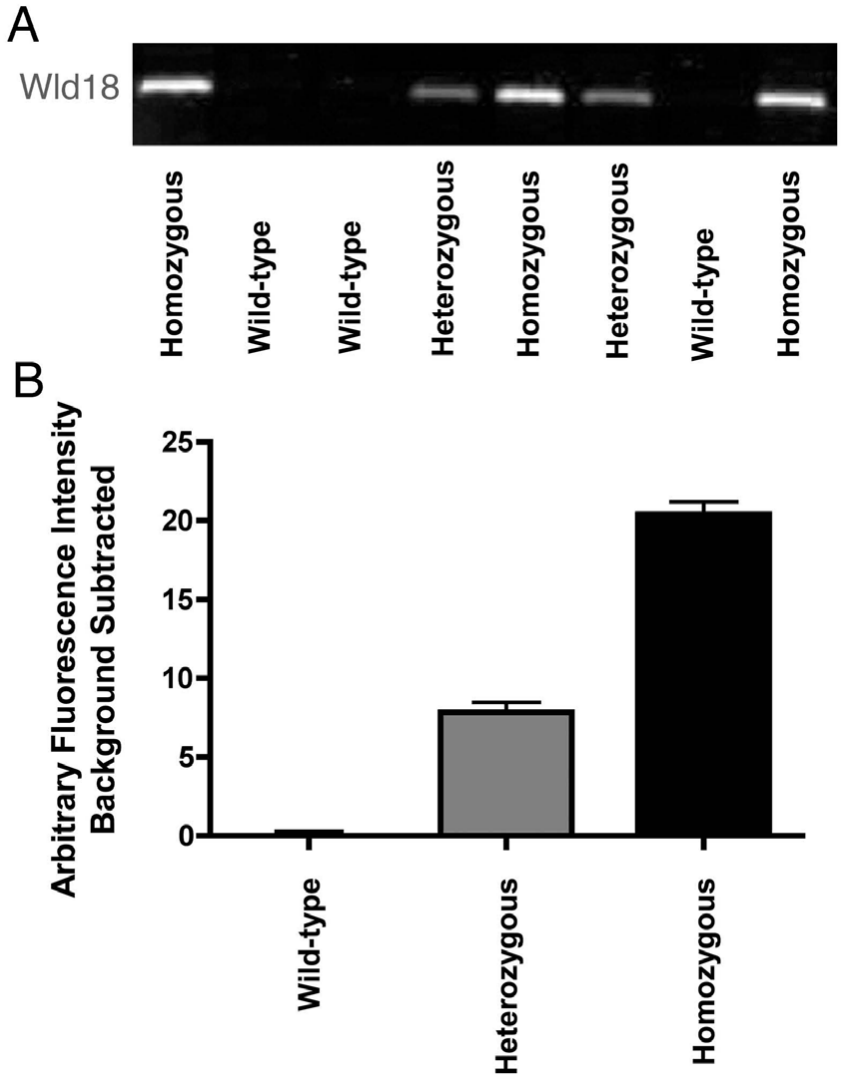
**Wld^S ^mice can be accurately genotyped by quantitative western blots of tail tip protein expression**. Tail tips were homogenized in RIPA buffer and following a BCA assay, 30 μg of protein were loaded per lane of a pre-cast gradient gel (see methods section). A - Representative example bands obtained with quantitative fluorescent western blots from a litter of mice produced by a heterozygous breeding pair. The litter contained wild-type, heterozygous and homozygous *Wld*^*S *^mice. Blots were probed for Wld^S ^protein levels using the Wld18 antibody. Tubulin was used as a loading control (data not shown). B - Wld^S ^protein expression shown as arbitrary fluorescence units, illustrating that protein expression was roughly doubled in homozygous mice compared to heterozygous mice. N = 4 wild-type mice, N = 3 heterozygous *Wld*^*S *^mice and N = 5 homozygous *Wld*^*S *^mice.

The observation that Wld^S ^protein is strongly expressed in non-neuronal tissues led us to investigate whether there would be sufficient protein in tail tips to facilitate accurate genotyping of *Wld*^*S *^mice using western blotting. We found that quantitative western blotting techniques easily distinguished between wild-type, heterozygous and homozygous *Wld*^*S *^mice (Figure 2).

### Systemic expression of *Wld*^*S *^did not affect gross appearance or growth rates

**Figure 3:**
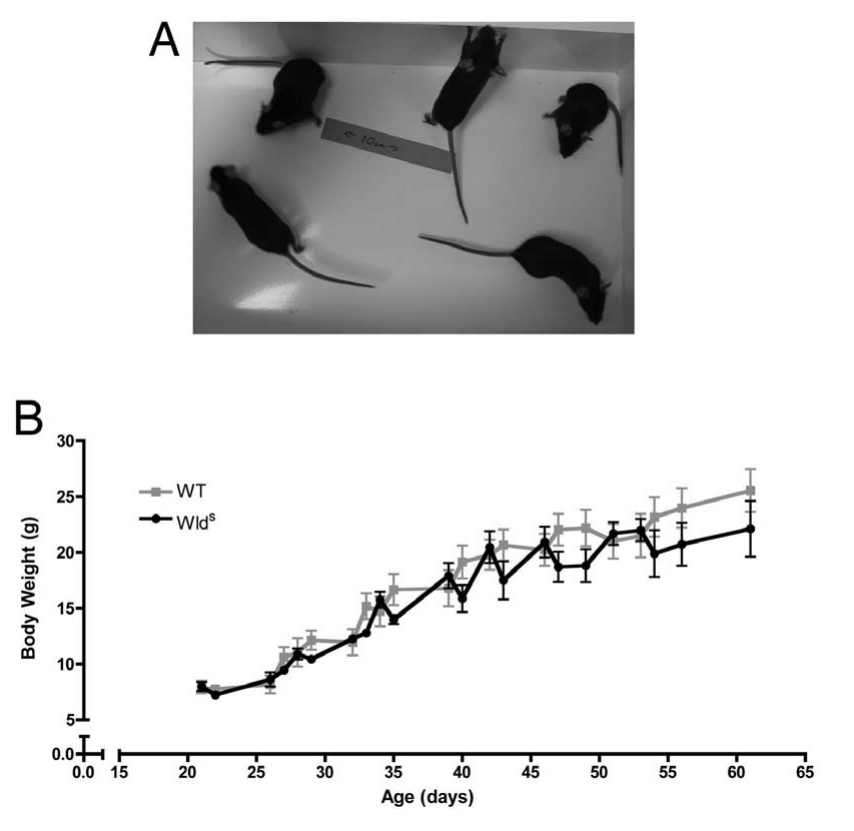
**Systemic expression of Wld^S ^does not affect gross appearance or growth rates of mice**. A - Photograph of 2 month old female mice from a representative litter produced by heterozygous *Wld*^*S *^breeding pairs. This litter contains wild-type, heterozygous and homozygous *Wld*^*S *^mice. There was no obvious gross difference in size, appearance or behaviour. B - Line graph showing body weight plotted against mouse age in days for wild-type (grey line) and *Wld*^*S *^(black line) mice. No significant differences were observed between genotypes at any time-point examined (Anova). N = 7 wild-type, N = 5 *Wld*^*S*^.

In order to test whether systemic *Wld*^*S *^expression affected total body weight, mice were bred from parents heterozygous for the *Wld*^*S *^mutation (see methods). Litters from this heterozygote cross contained mice which were null for *Wld*^*S *^(WT), mice which were heterozygous for *Wld*^*S *^(Het) and mice which were homozygous for *Wld*^*S *^(*Wld*^*S*^). All experimental comparisons were carried out within litters to remove any potential effects of background strain. There were no obvious qualitative differences in the size or behaviour of mice within litters. Figure 3A shows 5 female mice from the same experimental litter (containing a mixture of WT, Het and *Wld*^*S *^mice), indistinguishable from one another. Mice were weighed at regular intervals post weaning up to 2 months of age. There were no significant differences between the body weights of WT, Het and *Wld*^*S *^mice at any time-point examined (P > 0.2, ANOVA; Figure 3B; Het data not shown; N = 7 WT, N = 5 *Wld*^*S*^).

### *Wld*^*S *^expression did not affect gravimetrics or histopathology of non-neuronal tissue

**Figure 4:**
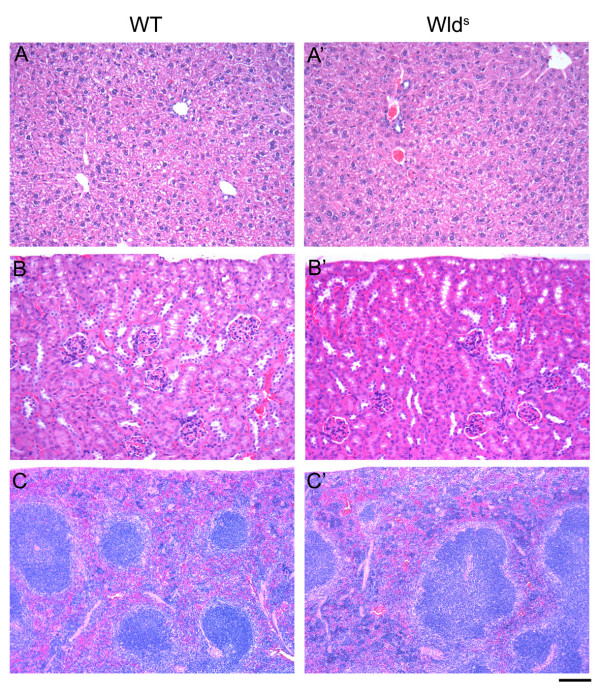
**Wld^S ^protein expression did not affect histopathology of non-neuronal organs**. H & E staining of tissues showed no obvious qualitative differences between wild type (left), heterozygous (data not shown) or homozygous *Wld*^*S *^mice (right) for liver (A), kidney (B), and spleen (C). The selected organs shown represent non-neuronal organs with low (liver), medium (kidney) and high (spleen) levels of Wld^S ^protein expression. Scale bar = 100 μm (A&B), 200 μm (C).

Detailed necropsies were carried out by an experienced rodent pathologist (DGB) on WT, Het and *Wld*^*S *^littermates to determine if non-neuronal expression of Wld^S ^protein had any affects on tissue gravimetrics (Table 1) or histopathology (Figure 4). Organs examined for gravimetrics included kidneys, liver, spleen, thymus, heart and whole brain. In order to minimize the effects of natural biological variability within litters data were expressed as organ weight per grams/body weight. No significant differences were observed between mice from any of the 3 genotypes (Table 1). Mice were also examined for alterations in body fat deposits (interscapular, pelvic, subcutaneous and mesenteric), with no evident differences (data not shown).

**Table 1:** Wld^S ^protein expression did not affect gravimetrics of non-neuronal organs (bwt: body-weight)

	WT Mean	WT SEM	Wld^S ^Mean	Wld^S ^SEM	t test
Bwt (g)	25.46	1.39	23.56	1.61	ns
Kidneys (g/bwt)	14.20	2.23	17.37	0.62	ns
Liver (g/bwt)	62.16	1.66	57.06	2.51	ns
Spleen (g/bwt)	3.73	0.25	4.24	0.44	ns
Thymus (g/bwt)	3.03	0.34	3.66	0.35	ns
Heart (g/bwt)	6.59	0.33	6.63	0.32	ns
Brain (g/bwt)	20.65	1.36	21.08	0.86	ns

Histopathological assessments were carried out on the following organs and tissues: kidney, heart, lungs, mediastinum, liver, gallbladder, urinary bladder, vagina, uterus, ovary, spleen, pancreas, lymph nodes, salivary gland, pituitary gland, adrenal gland, stomach, intestines (6 levels), calvarial bone, femur, tibia, knee joint, spine and spinal cord (4 levels, transverse sections), eyes, head, various sympathetic and parasympathetic ganglia, brain (transverse sections at 250 μm intervals). No significant differences were observed between any of the genotypes. Example sections for comparison are shown in Figure 4. Figure 4A shows representative images of liver from wild-type and *Wld*^*S *^mice, a tissue with little to no detectable Wld^S ^protein expression (see Figure 1). Note the presence of normal hepatocyte nuclei, sinusoids and central veins in both images. Figure 4B shows representative images of kidney cortex, a tissue with intermediate levels of Wld^S ^protein expression, showing the presence of normal glomeruli, Bowman's capsules and cortical tubular profiles. Figure 4C shows representative images of spleen, a tissue with high Wld^S ^protein expression, with normal red pulp and white pulp lymphoid accumulations.

### Cell cycle and cell stress alterations are specific to neuronal tissue

**Figure 5:**
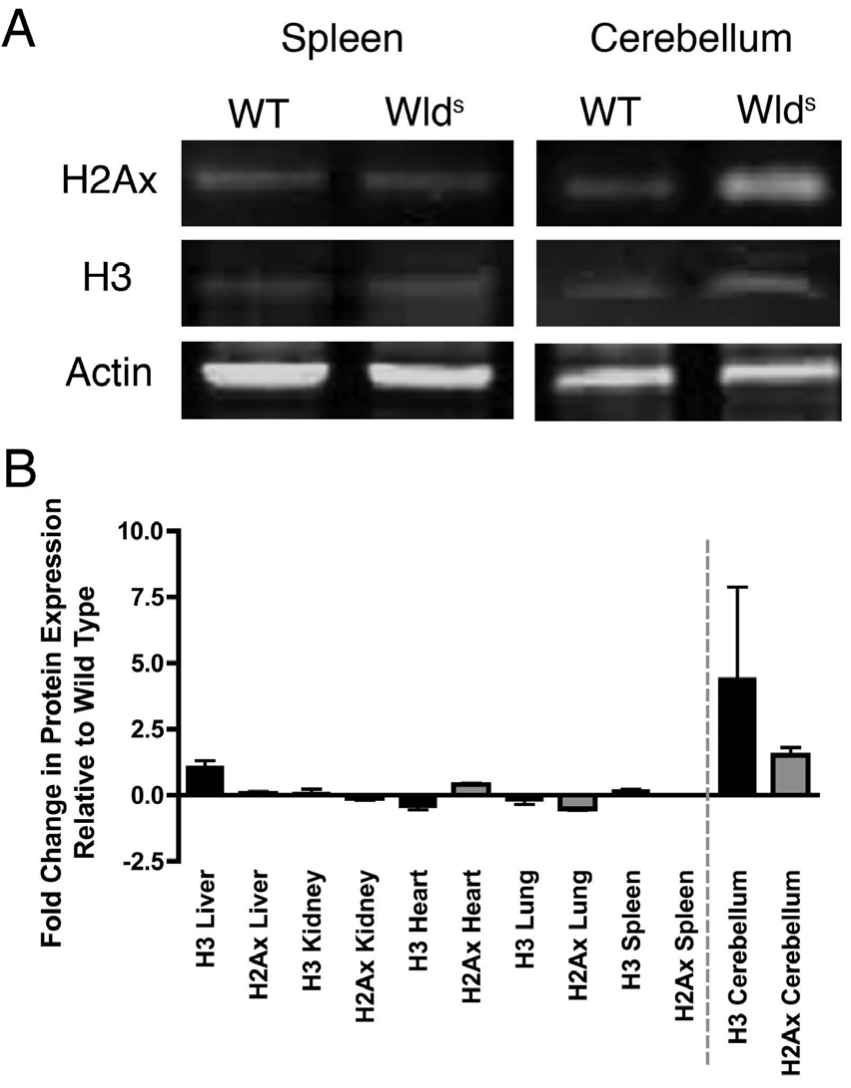
**Cell cycle and cell stress pathway alterations were specific to neuronal tissue**. A - Representative examples of bands obtained from quantitative fluorescent western blots from wild-type and *Wld*^*S *^mouse spleen and cerebellum probed with antibodies against cell stress (H2Ax) and cell cycle (H3) proteins (beta actin is shown as a loading control). Spleen was chosen because it had the highest Wld^S ^protein expression levels of all the non-neuronal tissues examined (Figure 1). Note the increases in H2Ax and H3 in *Wld*^*S *^cerebellum compared to wild-type mice, but no change in the expression of either protein in the spleen. B - Bar chart showing cell cycle (H3) and cell stress (H2Ax) protein levels in a range of organs from *Wld*^*S *^mice expressed as fold change in protein relative to wild-type. Both proteins examined showed only very minor fluctuations in expression levels in non-neuronal tissue (left hand side of dotted line). The magnitude of change in non-neuronal tissue did not begin to approach those observed in the cerebellum of *Wld*^*S *^mice (right hand side of dotted line). A minimum of 3 mice per genotype were used for each experiment.

We have previously reported alterations in proteins involved with cell cycle and cell stress in non-injured neural tissue from *Wld*^*S *^mice *in vivo *and *in vitro *[26,27]. In order to determine whether similar changes were instigated in non-neuronal organs and tissues expressing Wld^S ^protein we examined expression levels of the cell stress marker antiphosphohistone H2Ax (H2Ax) and a marker of cell cycle progression acetylated histone H3 (H3) in liver, kidney, heart, lung, spleen and cerebellum from wild-type and *Wld*^*S *^mice. Example bands for both of these proteins are shown in Figure 5A. As previously demonstrated there was a significant (P < 0.001; unpaired t-test) increase in expression of both cell stress and cell cycle markers in *Wld*^*S *^cerebellum compared to wild-type (Figure 5). However, no changes in expression of either of these proteins were found in any of the non-neuronal organs examined (Figure 5). Thus, previously reported differences in cell cycle and cell stress status appear to be a neuronal-specific response to expression of *Wld*^*S *^at a basal level.

## Discussion

Here we have shown that *Wld*^*S *^RNA and protein are expressed at differing levels in a range of non-neuronal organs in *Wld*^*S *^mice but did not influence overall body weight or growth. Gravimetric and histological analysis of a wide range of organs demonstrated that the presence of *Wld*^*S *^RNA and/or protein had no overt influence on non-neuronal tissues at a basal level. We have also shown that previously reported alterations in cell cycle and cell stress proteins reported in *Wld*^*S *^brain tissue are neuronal-specific and are not observed in non-neuronal organs *in vivo*. It will now be of interest to establish whether Wld^S ^protein can modify responses to injury in non-neuronal tissues and organs, where it is expressed. Expression data in the current study show that the spleen has a comparatively high level of protein, suggesting that this organ may be useful for such studies.

These data demonstrate that downstream consequences of *Wld*^*S *^expression, incorporating pathways including NAD biosynthesis, ubiquitination, the mitochondrial proteome, cell cycle status and cell stress [17,26-28], do not have any major adverse effects on non-neuronal organs. This suggests that the utilization of *Wld*^*S*^-based therapeutics for treating neurodegenerative conditions can be considered safe for other body systems, should the treatment spread beyond the confines of the nervous system (e.g. unintentionally or as a result of systemic administration). Studies reporting that *Wld*^*S*^-mediated neuroprotection (as well as many of its downstream effectors such as Nmnat pathways) can be successfully delivered to the nervous system using approaches such as viral gene delivery suggest that *Wld*^*S*^-based therapeutics are a realistic possibility [16,17].

Our experimental data have also revealed nervous system-specific effects of *Wld*^*S *^expression. The finding that modifications in cell cycle and cell stress status previously reported in neurons expressing *Wld*^*S *^[27] are not replicated in non-neuronal tissues suggests that neurons may have distinct intrinsic responses to the presence of *Wld*^*S*^. One possible explanation for this is that neurons are terminally differentiated cells whose cell cycle status is markedly different from many non-neuronal cells (for review see [32]). Whether or not this contributes directly to the neuroprotective phenotype remains to be determined. Nevertheless, these data support the hypothesis that *Wld*^*S *^acts by targeting a specific step(s) in degenerative pathways intrinsic to neurons [33].

## Conclusions

We conclude that expression of Wld^S ^protein has no adverse effects on non-neuronal tissue at a basal level *in vivo*, supporting the possibility of its safe use in future therapeutic strategies targeting axonal and/or synaptic compartments in patients with neurodegenerative disease.

## Methods

### Mouse breeding

Natural mutant C57Bl6/Wld^S ^(*Wld*^*S*^) mice and C57Bl/6 (wild-type) mice were obtained from Harlan Olac Laboratories (Bicester, UK) and housed within the animal care facilities in Edinburgh. All animal experiments were carried out in accordance with the guidance and rules of, and under license from, the UK Home Office. Breeding pairs made up of one *Wld*^*S *^mouse and one wild-type mouse were used to generate heterozygous *Wld*^*S *^mice. These mice were then bred to produce litters which contained mice null for the *Wld*^*S *^mutation (wild-type), mice heterozygous for the mutation (Het) and mice homozygous for the mutation (*Wld*^*S*^). All data were obtained from tissue harvested from 1-2 month old mice. For body weight measurements, mice were weighed every 2 days post weaning. A minimum of 3 mice were used per group for all experiments.

### Genotyping

Mice were bred as detailed above, ear-notched and assigned individual identifying numbers which were used so the experimenter remained blind to the genotype of individual mice throughout. Mice were genotyped post mortem and their genetic status was only assigned after data collection and analysis was complete. Mice were initially genotyped by quantitative western blotting for Wld^S ^protein expression levels using protein extracted from tail tips using similar methodology described below (see Figure 2). These genotypes were all validated by real-time PCR as previously described [34].

### Necropsy

Mice were killed with carbon dioxide gas and immediately weighed. Selected organs were weighed and a standard panel of organs were immersion-fixed in 10% neutral-buffered formalin for histopathology. Fixed organs were embedded in paraffin, sectioned at 4 mm, and stained with hematoxylin and eosin. All analyses were undertaken with the investigator blind to the genotype of each animal.

### RNA extraction & qRT-PCR

Cerebellum, liver, spleen, thymus, lung, and heart from 6-week-old female *Wld*^*S *^mice were flash frozen on dry ice and mRNA was extracted using the RNAEasy Kit (Qiagen, Valencia, CA). Messenger RNA from all tissues was transformed into cDNA using the Superscript III kit (Invitrogen, Carlsbad, CA). Quantitative real-time PCR (qRT-PCR) was performed to examine *Wld*^*S *^gene expression using a Sybr-Green '1-step qRT-PCR kit' (Invitrogen) on an ABI PRISM 7700 Instrument (Applied Biosystems, Foster City, CA). The following primer sequences were used:

Wld^S^-726F TGTGCCCAAGGTGAAATTGC

Wld^S^-818R ACGATTTGCGTGATGTCCTCC

β-*actin *was used as a control gene. To verify that there was minimal genomic DNA contamination, we also performed qRT-PCR analysis of selected extracted mRNA samples prior to conversion to cDNA, which demonstrated a negligible genomic DNA presence (10,000× less).

### Protein extraction & quantitative western blotting

Mice were killed by cervical dislocation and organs for examination rapidly removed. Protein was extracted from tail tips, cerebella and organs including the kidney, liver, heart, lung and spleen of age- and sex-matched mice in RIPA buffer with 10% protease inhibitor cocktail (Sigma) as previously described [26,27]. 30 μg of protein per lane was separated by SDS/Polyacrylamide gel electrophoresis on 4-20% pre-cast NuPage 4-12% Bis Tris gradient gels (Invitrogen) and then transferred to PVDF membrane overnight. The membranes were then blocked using Odyssey blocking buffer (Li-COR) and incubated with primary antibodies as per manufacturers instructions (anti acetyl Histone H3 - Lake Placid Biologicals; antiphosphohistone H2Ax - Upstate; anti beta actin and anti beta-III-tubulin - Abcam). Wld-18 antibodies were a kind gift from Dr Michael Coleman and were used as previously described [30]. Odyssey secondary antibodies were added according to manufacturers instructions (Goat anti rabbit IRDye 680 and Goat anti mouse IRDye 800). Blots were imaged using an Odyssey Infrared Imaging System (Li-COR Biosciences). Scan resolution of the instrument ranges from 21-339 μm and in this study blots were imaged at 169 μm. Quantification was performed on single channels with the Li-COR analysis software provided, as previously described [26,27].

## Authors' contributions

TMW participated in the design of the study, carried out experiments, analysed data and drafted the manuscript. DGB carried out necropsy experiments and analysed data. DT carried out experiments. AMT/KMB/JWT carried out QPCR experiments. THG conceived of the study, participated in its design and coordination, analysed data and drafted the manuscript. All authors read and approved the final manuscript.

## References

[B1] Raff MC Whitmore AV Finn JT Axonal self-destruction and neurodegeneration Science 2002 296 868 871 10.1126/science.1068613 11988563

[B2] Wishart TM Parson SH Gillingwater TH Synaptic vulnerability in neurodegenerative disease J Neuropathol Exp Neurol 2006 65 733 739 10.1097/01.jnen.0000228202.35163.c4 16896307

[B3] Selkoe DJ Alzheimer's disease is a synaptic failure Science 2002 298 789 791 10.1126/science.1074069 12399581

[B4] Partanen S Haapanen A Kielar C Pontikis C Alexander N Inkinen T Saftig P Gillingwater TH Cooper JD Tyynelä J Synaptic changes in the thalamocortical system of cathepsin D-deficient mice: A model of human congenital neuronal ceroid-lipofuscinosis J Neuropathol Exp Neurol 2008 67 16 29 10.1097/nen.0b013e31815f3899 18091563

[B5] Kielar C Wishart TM Palmer A Dihanich S Wong AM Macauley SL Chun C-H Sands MS Pearce D Cooper JD Gillingwater TH Molecular correlates of axonal and synaptic pathology in mouse models of Batten disease Hum Mol Genet 2009 18 4066 4080 10.1093/hmg/ddp355 19640925 PMC2758138

[B6] Trapp BD Peterson J Ransohoff RM Rudick R Mörk S Bö L Axonal transection in the lesions of multiple sclerosis N Engl J Med 1998 338 278 285 10.1056/NEJM199801293380502 9445407

[B7] Fischer LR Culver DG Tennant P Davis AA Wang M Castellano-Sanchez A Khan J Polak MA Glass JD Amyotrophic lateral sclerosis is a distal axonopathy: evidence in mice and man Exp Neurol 2004 185 232 240 10.1016/j.expneurol.2003.10.004 14736504

[B8] Murray LM Comley LH Thomson D Parkinson N Talbot K Gillingwater TH Selective vulnerability of motor neurons and dissociation of pre- and post-synaptic pathology at the neuromuscular junction in mouse models of spinal muscular atrophy Hum Mol Genet 2008 17 949 962 10.1093/hmg/ddm367 18065780

[B9] Lunn ER Perry VH Brown MC Rosen H Gordon S Absence of Wallerian degeneration does not hinder regeneration in peripheral nerve Eur J Neurosci 1989 1 27 33 10.1111/j.1460-9568.1989.tb00771.x 12106171

[B10] Gillingwater TH Ingham CA Parry KE Wright AK Haley JE Wishart TM Arbuthnott GW Ribchester RR Delayed synaptic degeneration in the CNS of Wld^*S *^mice after cortical lesion Brain 2006 129 1546 1556 10.1093/brain/awl101 16738060

[B11] Sajadi A Schneider BL Aebischer P Wlds-mediated protection of dopaminergic fibres in an animal model of Parkinson disease Curr Biol 2004 14 326 330 14972684 10.1016/j.cub.2004.01.053

[B12] Hasbani DM O'Malley KL Wld^S ^mice are protected against the Parkinsonian mimetic MPTP Exp Neurol 2006 202 93 99 10.1016/j.expneurol.2006.05.017 16806180

[B13] Samsam M Mi W Wessig C Zielasek J Toyka KV Coleman MP Martini R The Wlds mutation delays robust loss of motor and sensory axons in a genetic model for myelin-related axonopathy J Neurosci 2003 23 2833 2839 12684470 10.1523/JNEUROSCI.23-07-02833.2003 PMC6742108

[B14] Ferri A Sanes JR Coleman MP Cunningham JM Kato AC Inhibiting axon degeneration and synapse loss attenuates apoptosis and disease progression in a mouse model of motorneurone disease Curr Biol 2003 13 669 673 10.1016/S0960-9822(03)00206-9 12699624

[B15] Gillingwater TH Haley JE Ribchester RR Horsburgh K Neuroprotection after transient global cerebral ischaemia in Wld^*S *^mutant mice J Cereb Blood Flow Metab 2004 24 62 66 10.1097/01.WCB.0000095798.98378.34 14688617

[B16] Wang MS Fang G Culver DG Davis AA Rich MM Glass JD The Wld^*S *^protein protects against axonal degeneration: a model of gene therapy for peripheral neuropathy Ann Neurol 2001 50 773 779 10.1002/ana.10039 11761475

[B17] Araki T Sasaki Y Milbrandt J Increased nuclear NAD biosynthesis and SIRT1 activation prevent axonal degeneration Science 2004 305 1010 1013 10.1126/science.1098014 15310905

[B18] Coleman MP Conforti L Buckmaster EA Tarlton A Ewing RM Brown CM Lyone MF Perry VH An 85-Kb tandem triplication in the slow Wallerian degeneration (Wld^S^) mouse Proc Natl Acad Sci USA 1998 95 9985 9990 10.1073/pnas.95.17.9985 9707587 PMC21448

[B19] Mi W Conforti L Coleman MP The slow Wallerian degeneration mutation (Wld^S^): genotyping methods and mutation stability studies FENS Forum Session 225-Trauma 2002 Abstract 225.2

[B20] Mi W Glass JD Coleman MP Stable inheritance of an 85 Kb triplication in C57BL/Wld^*S *^mice Mutation Res 2003 526 33 37 12714180 10.1016/s0027-5107(03)00011-3

[B21] Lyon MF Ogunkolade BW Brown MC Atherton DJ Perry VH A gene affecting Wallerian nerve degeneration maps distally on mouse chromosome 4 Proc Natl Acad Sci USA 1993 90 9717 9720 10.1073/pnas.90.20.9717 8415768 PMC47641

[B22] Conforti L Tarlton A Mack TG Mi W Buckmaster EA Wagner D Perry VH Coleman MP A Ufd2/D4Cole1e chimeric protein and overexpression of Rbp7 in the slow Wallerian degeneration (Wld^S^) mouse Proc Natl Acad Sci USA 2000 97 11377 11382 10.1073/pnas.97.21.11377 11027338 PMC17208

[B23] Mack TGA Reiner M Beirowski B Mi W Emanuelli M Wagner D Thomson D Gillingwater T Court F Conforti L Shama Fernando F Tarlton A Andressen C Addicks K Magni G Ribchester RR Perry VH Coleman MP Wallerian degeneration of injured axons and synapses is delayed by a Ube4b/Nmnat chimeric gene Nat Neurosci 2001 4 1199 1206 10.1038/nn770 11770485

[B24] Adalbert R Gillingwater TH Haley JE Bridge K Beirowski B Berek L Wagner D Grumme SG Thomson D Addicks K Ribchester RR Coleman MP A rat model of slow Wallerian degeneration (Wld^S^) with improved preservation of neuromuscular synapses Eur J Neurosci 2005 21 271 277 10.1111/j.1460-9568.2004.03833.x 15654865

[B25] MacDonald JM Beach MG Porpiglia E Sheehan AE Watts RJ Freeman MR The Drosophila cell corpse engulfment receptor Draper mediates glial clearance of severed axons Neuron 2006 50 869 881 10.1016/j.neuron.2006.04.028 16772169

[B26] Wishart TM Paterson JM Short DM Meredith S Robertson KA Sutherland C Cousin MA Dutia MB Gillingwater TH Differential proteomics analysis of synaptic proteins identifies potential cellular targets and protein mediators of synaptic neuroprotection conferred by the slow Wallerian degeneration (Wlds) gene Mol Cell Proteomics 2007 6 1318 1330 10.1074/mcp.M600457-MCP200 17470424 PMC2225590

[B27] Wishart TM Pemberton HN James SR McCabe CJ Gillingwater TH Modified cell cycle status in a mouse model of altered neuronal vulnerability (Wallerian Degeneration Slow; *Wld*^*S*^) Genome Biol 2008 9 6 R101 10.1186/gb-2008-9-6-r101 18570652 PMC2481432

[B28] Yahata N Yuasa S Araki T Nicotinamide mononucleotide adenylyltransferase expression in mitochondrial matrix delays Wallerian degeneration J Neurosci 2009 29 6276 6284 10.1523/JNEUROSCI.4304-08.2009 19439605 PMC6665489

[B29] Gillingwater TH Wishart TM Chen PE Haley JE Robertson K MacDonald SH-F Middleton S Wawrowsky K Shipston MJ Melmed S Wyllie DJA Skehel PA Coleman MP Ribchester RR The neuroprotective Wld^*S *^gene regulates expression of PTTG1 and erythroid differention regulator 1-like gene in mice and human cells Hum Mol Genet 2006 15 625 635 10.1093/hmg/ddi478 16403805

[B30] Wilbrey A Haley J Wishart T Conforti L Morreale G Beirowski B Babetto E Adalbert R Gillingwater TH Smith T Wyllie DJA Ribchester RR Coleman MP VCP binding influences intracellular distribution of the slow Wallerian degeneration protein, Wld^S^ Mol Cell Neurosci 2008 38 325 340 10.1016/j.mcn.2008.03.004 18468455

[B31] Fang C Bernardes-Silva M Coleman MP Perry VH The cellular distribution of the Wld^S ^chimeric protein and its constituent proteins in the CNS Neuroscience 2005 135 1107 1118 10.1016/j.neuroscience.2005.06.078 16154290

[B32] Herrup K Yang Y Cell cycle regulation in the postmitotic neuron: oxymoron or new biology? Nat Rev Neurosci 2007 8 368 378 10.1038/nrn2124 17453017

[B33] Perry VH Brown MC Lunn ER Tree P Gordon S Evidence that very slow Wallerian degeneration in C57BL/Ola mice is an intrinsic property of the peripheral nerve Eur J Neurosci 1990 2 802 808 10.1111/j.1460-9568.1990.tb00472.x 12106282

[B34] Wishart TM MacDonald SHF Chen PE Shipston MJ Coleman MP Gillingwater TH Ribchester RR Design of a novel quantitative PCR (QPCR)-based protocol for genotyping mice carrying the neuroprotective Wallerian degeneration slow (*Wld*^*S*^) gene Mol Neurodegen 2007 2 21 10.1186/1750-1326-2-21 PMC2147001 17971231

